# How Safe Is Our Food?

**DOI:** 10.3201/eid1701.101821

**Published:** 2011-01

**Authors:** J. Glenn Morris

**Affiliations:** Author affiliation: University of Florida, Gainesville, Florida, USA

**Keywords:** Foodborne disease, disease burden, food attribution, United States, commentary

How safe is our food? Put another way, how much illness in the United States is caused by foodborne pathogens? It sounds like a simple question. Getting a reasonable answer, however, is far from simple. The basic problem lies in the fact that only a small fraction of foodborne disease cases get reported through official (or unofficial) reporting systems. Calculating the “real” rate of foodborne illness requires development of models that use reported cases as a starting point to estimate underlying disease rates. Given the plethora of pathogens that can be transmitted through foodborne routes, this is a complex, and somewhat daunting, process. It is, however, necessary for assessing the safety of foods and developing strategies for disease prevention. The articles by Scallan et al. (*1*,*2*) in this issue represent the latest efforts to develop such estimates of the magnitude of foodborne illness in the United States.

In 1999, Mead et al. (*3*) published initial estimates of foodborne disease in the United States. This landmark undertaking was the first to provide a comprehensive compilation of data from a variety of sources, including the Centers for Disease Control and Prevention (CDC) and the medical literature. It resulted in the often-cited estimates that foodborne pathogens cause 76 million episodes of illness, 325,000 hospitalizations, and 5,000 deaths each year in the United States. (Hereafter, episodes of illness are referred to as illnesses.) During the past decade, these numbers have strongly driven ongoing efforts to implement or reform regulatory systems to protect the public from foodborne illness. However, some aspects of the methods have been criticized, particularly the high degree of uncertainty of particular parameters and thus of the results themselves (*4**–**6*). These concerns have led to requests for CDC to repeat and update the work of Mead et al., using better methods and parameter estimates that more closely reflect current realities.

Now, ≈11 years later, Scallan et al. have produced “Sons of Mead,” which include substantial improvements to the methods used by Mead et al. and to the quality and timeliness of data (*1*,*2*). Scallan et al. should be commended, especially for 2 specific improvements: their advanced treatment of statistical uncertainty and variability and their transparent inclusion of voluminous appendixes of data, models, and assumptions. These authors followed the same basic approach as Mead et al. but chose to report their estimates in 2 articles. In the first article, they based their estimates of illnesses caused by 24 major pathogens (e.g., *Salmonella* spp., *Escherichia coli* O157:H7) primarily on data from the Foodborne Diseases Active Surveillance Network (FoodNet) and other pathogen-specific surveillance systems. In the second article, they estimated illnesses caused by unknown (or unspecified) pathogens by subtracting illnesses caused by known pathogens from the annual estimated number of cases of acute gastroenteritis in the US population and adjusting the result by the percentage assumed to be acquired domestically through food. If these 2 estimates are combined, as they were by Mead et al., the new totals are 47.8 million foodborne illnesses, 127,839 hospitalizations, and 3,037 deaths per year in the United States.

When one compares the 1999 and 2010 estimates (76 million vs. 47.8 million illnesses), the immediate response is to ask: Does this mean that food in this country is safer than it was 11 years ago? Unfortunately, the Scallan et al. articles do not enable us to answer this question. The methods, underlying assumptions, and parameter estimates used to generate these new numbers differ sufficiently from those used ≈11 years ago to preclude comparisons. In fact, if one looks simply at rates of overall gastrointestinal illness in the United States, based on FoodNet Population Surveys (*2*), one might infer that overall rates of acute gastrointestinal illness have increased during this period, from 0.49 episodes per person per year in 2000–2001, to 0.54 in 2002–2003, and to 0.73 in 2006–2007 (see [*7*] for a discussion of some methodologic issues with regard to the 2006–2007 survey). For the Scallan et al. articles, these 3 numbers were averaged to arrive at a rate of 0.6 episodes of acute gastroenteritis per person per year over the past decade. In contrast, Mead et al. used an estimate of 0.79 episodes of gastroenteritis per person per year, based on FoodNet data but also on older community surveys; they also used a somewhat different definition of acute gastrointestinal illness. This difference in estimated annual rates of acute gastroenteritis, when combined with a lower assumed proportion of gastroenteritis that is foodborne, explains much of the dramatic drop in total annual episodes of foodborne disease. Had Scallan et al. elected to use the 2006–2007 FoodNet estimate of 0.73 cases per person per year rather than use the average of 0.6 cases, their numbers would have been substantially higher and closer to the Mead et al. estimates.

Thus, if we can’t use the Scallan estimates for comparison, is there any way to say whether food in the United States is safer now than it was 11 years ago? The best answer to this question comes from the FoodNet system (*7*), an active laboratory-based sentinel surveillance system that was established to monitor the public health impact of the 1995 US Department of Agriculture (USDA) Pathogen Reduction: Hazard Analysis and Critical Control Point (HAACP) System regulations (the first major revision of USDA food safety regulations since 1906). FoodNet provides annual data from designated sentinel surveillance sites on numbers of laboratory-diagnosed cases of 10 predominantly foodborne bacterial and parasitic pathogens; it reports actual case totals, not estimates. Despite year-to-year variability (including significant decreases in incidence of *Shigella* spp. and *E. coli* O157:H7 for 2009) (*8*), the overall trends show an initial drop in incidence of infection with the major bacterial foodborne pathogens after implementation of the 1995 USDA regulations, followed by a leveling off of incidence in subsequent years. One exception is infections caused by *Vibrio* spp., which are increasing, partly because climate change is affecting coastal environments (*9*). Bottom line: with the exception of *Vibrio* spp., things don’t seem to be getting worse; however, after the initial decline since the USDA regulatory changes in 1995, one does not see evidence of sustained improvement.

How do numbers from the United States compare with those from Europe and the rest of the world? Again, differences in methods used by Scallan et al. make it difficult, if not impossible, to directly compare these numbers with those being published by other countries, including Canada, Australia, and members of the European Union (*10**–**12*). Although these new estimates cannot be compared directly with previous estimates or with estimates from other countries, these articles nonetheless constitute a necessary starting point for generation of more robust and regularly updated numbers. Looking across time, use of a consistent method, with regular updating of data (ideally annually), would provide a basis for assessing the effect of changes in regulation and other interventions at a national level. Similarly, if the methods are further modified in keeping with current international discussions on standardization of foodborne disease estimates (*13*), direct comparison of US numbers with those from other countries may become possible.

Estimates of the relative burden of disease caused by specific pathogens are crucial for improving our understanding of foodborne illness risks, but they are insufficient on their own. To target interventions (which are almost always food specific), illnesses must be quantified in terms of food–pathogen combinations. Doing so, in turn, requires development of what have been termed food attribution data (*14**,**15*). That is, how much salmonellosis is caused by eating contaminated chicken versus eggs, beef, or pork? How often is beef, compared with produce, the source of infection with *E. coli* O157:H7? Likewise, summary statistics such as number of cases, hospitalizations, and deaths ignore at-risk subpopulations and chronic sequelae such as end-stage renal disease, congenital toxoplasmosis, and irritable bowel syndrome. As such, the World Health Organization and many industrialized countries are increasingly reporting integrated measures of disease, such as disability-adjusted life years, which more fully capture disease symptoms and severities (*13*). Furthermore, to reduce specific foodborne hazards, we need information about the many factors along the complex farm-to-table pathway that can lead to the introduction or amplification of pathogens that contaminate food. This information would also help determine feasibility and efficacy of potential interventions.

As outlined in a recent Institute of Medicine report (*16*), implementation of a modern, risk-based food safety system in the United States will ultimately require much better data and a strong analytic capacity at the federal level that cuts across current agency lines. Although we still have a long way to go to bring our food safety system into the current century, the articles by Scallan et al. are critical steps in the right direction.

## References

[1] Scallan E, Hoekstra RM, Angulo FJ, Tauxe RV, Widdowson M-A, Roy SL, Foodborne illness acquired in the United States—major pathogens. Emerg Infect Dis. 2010;17:7–15. 10.3201/eid1701.P11101 PMC3375761 21192848

[2] Scallan E, Griffin PM, Angulo FJ, Tauxe RV, Hoekstra RM. Foodborne illness acquired in the United States—unspecified agents. Emerg Infect Dis. 2011;17:16–22. 21192849 10.3201/eid1701.P21101 PMC3204615

[3] Mead PS, Slutsker L, Dietz V, McCaig LF, Bresee JS, Shapiro C, Food-related illness and death in the United States. Emerg Infect Dis. 1999;5:607–24. 10.3201/eid0505.990502 10511517 PMC2627714

[4] Powell M, Ebel E, Schlosser W. Considering uncertainty in comparing the burden of illness due to foodborne microbial pathogens. Int J Food Microbiol. 2001;69:209–15. 10.1016/S0168-1605(01)00495-0 11603858

[5] Phillips CV, LaPole LM. 2003. Quantifying errors without random sampling. BMC Medical Research Methodology 2003;3:9 [cited 2010 Nov 15]. http://www.biomedcentral.com/1471-2288/3/9 10.1186/1471-2288-3-9 PMC166164 12892568

[6] Frenzen PD. Deaths due to unknown foodborne agents. Emerg Infect Dis. 2004;10:1536–43. 15498153 10.3201/eid1009.030403 PMC3320286

[7] Cantwell LB, Henao OL, Hoekstra RM, Scallan E. The effect of different recall periods on estimates of acute gastroenteritis in the United States, FoodNet Population Survey 2006–2007. Foodborne Pathog Dis. 2010;7:1225–8. 10.1089/fpd.2010.0567 20578914

[8] Centers for Disease Control and Prevention. Preliminary FoodNet data on the incidence of infection with pathogens transmitted commonly through food—10 states, 2009. MMWR Morb Mortal Wkly Rep. 2010;59:418–22. 20395935

[9] Lipp EK, Huq A, Colwell RR. Effects of global climate on infectious disease: the cholera model. Clin Microbiol Rev. 2002;15:757–70. 10.1128/CMR.15.4.757-770.2002 12364378 PMC126864

[10] Adak GK, Long SM, O’Brien SJ. Trends in indigenous foodborne disease and deaths, England and Wales: 1992–2000. Gut. 2002;51:832–41. 10.1136/gut.51.6.832 12427786 PMC1773489

[11] OzFoodNet Net Working Group. Monitoring the incidence and causes of diseases potentially transmitted by food in Australia: annual report of the OzFoodNet Network, 2008. Commun Dis Intell. 2009;33:389–413. 20301968 10.33321/cdi.2009.33.42

[12] Flint JA, Van Duynhoven YT, Angulo FJ, DeLong SM, Braun P, Kirk M, Estimating the burden of acute gastroenteritis, foodborne disease, and pathogens commonly transmitted by food: an international review. Clin Infect Dis. 2005;41:698–704. 10.1086/432064 16080093

[13] Kuchenmuller T, Hird S, Stein C, Kramarz P, Nanda A, Havelaar AH. Estimating the global burden of foodborne diseases—a collaborative effort. Euro Surveill. 2009;14:pii:19195. 10.2807/ese.14.18.19195-en 19422776

[14] Batz MB, Doyle MP, Morris JG, Painter J, Singh R, Tauxe RV, ; Food Attribution Working Group. Linking illness to food: summary of a workshop on food attribution. Emerg Infect Dis. 2005;11:993–9. 16022770 10.3201/eid1107.040634 PMC3371809

[15] Pires SM, Evers EE, van Pelt W, Ayers T, Scallan E, Angulo FJ, ; Med-Vet-Net Workpackage 28 Working Group. Attributing the human disease burden of foodborne infections to specific sources. Foodborne Pathog Dis. 2009;6:417–24. 10.1089/fpd.2008.0208 19415971

[16] Institute of Medicine. Enhancing food safety: the role of the Food and Drug Administration. Washington: National Academy Press; 2010. p. 1–576.

